# The *Carbon_h-*Factor: Predicting Individuals' Research Impact at Early Stages of Their Career

**DOI:** 10.1371/journal.pone.0028770

**Published:** 2011-12-14

**Authors:** Claus-Christian Carbon

**Affiliations:** Department of General Psychology and Methodology, University of Bamberg, Bamberg, Germany; Science and Technology Facilities Council, United Kingdom

## Abstract

Assessing an individual's research impact on the basis of a transparent algorithm is an important task for evaluation and comparison purposes. Besides simple but also inaccurate indices such as counting the mere number of publications or the accumulation of overall citations, and highly complex but also overwhelming full-range publication lists in their raw format, Hirsch (2005) introduced a single figure cleverly combining different approaches. The so-called *h*-index has undoubtedly become the standard in scientometrics of individuals' research impact (note: in the present paper I will always use the term “research impact” to describe the research performance as the logic of the paper is based on the *h*-index, which quantifies the specific “impact” of, e.g., researchers, but also because the genuine meaning of impact refers to quality as well). As the *h*-index reflects the number *h* of papers a researcher has published with at least *h* citations, the index is inherently positively biased towards senior level researchers. This might sometimes be problematic when predictive tools are needed for assessing young scientists' potential, especially when recruiting early career positions or equipping young scientists' labs. To be compatible with the standard *h*-index, the proposed index integrates the scientist's research age (*Carbon_h*-factor) into the *h*-index, thus reporting the average gain of *h*-index per year. Comprehensive calculations of the *Carbon_h*-factor were made for a broad variety of four research-disciplines (economics, neuroscience, physics and psychology) and for researchers performing on three high levels of research impact (*substantial*, *outstanding* and *epochal*) with ten researchers per category. For all research areas and output levels we obtained linear developments of the *h*-index demonstrating the validity of predicting one's later impact in terms of research impact already at an early stage of their career with the *Carbon_h*-factor being approx. 0.4, 0.8, and 1.5 for substantial, outstanding and epochal researchers, respectively.

## Introduction

Assessing an individual's research impact on the basis of a transparent algorithm is an important task for evaluation and comparison purposes as this method abstracts from individually used and interpreted facts. This importance will continuously grow due to increasing research costs [1] while scientific resources are strongly limited [2], [3]. The field of scientometrics has developed a large repertoire of possible measures, some of which are rather simple but also very limited in their validity. Others are too complex or too overwhelming for the single evaluator. Simple measures are, inter alia, the number of publications, which might not be a valid measure of quality due to the possibility of researchers overemphasising quantity versus quality. The vast amount of output, thus, should never be a criterion of quality [4], [5]. The overall number of citations is also vulnerable to invalid measurement as the distribution of citations across different publications is not taken into account. The integration of multidimensional sources of evidence for research impact (note: in the present paper I will always use the term “research impact” to describe the research performance as the logic of the paper is based on the *h*-index, which quantifies the specific “impact” of, e.g., researchers, but also because the genuine meaning of impact refers to quality as well), such as the number and scope of research projects, academic prizes, prestigious keynotes and being a trigger for influential public debates, would be helpful, but appears to be impracticable. First, such evaluations often simply overwhelm the evaluator, second, such information can hardly be obtained to full extent, third, any evaluation on such a basis is likely to be incommensurable, and thus cannot be compared among different candidates. Taking these difficulties into account, Hirsch [4] proposed a single index which is a) simple to understand, b) simple to calculate, c) transparent and d) easy to compare among different researchers. Consequently, the so-called *h*-index has gained widespread popularity as a measure for comparing researchers' output [6]. It is now, as it is being incorporated as a regular part of several research information platforms [7],[8], such as ISI Web of Science by Thomson Reuters or Scopus by Elsevier B.V., in fact the standard measure for assessing research impact (cf. [9], [10]).

Hirsch ([4], p. 16569]) defined the *h*-index in his seminal paper as follows: “A scientist has index *h* if *h* of his/her *N_p_* papers have at least *h* citations each, and the other (*N_p_*−*h*) papers have no more than *h* citations each.” Evidently, this simple calculation on basis of the number of papers and citations has an important drawback when young scientists' performances have to be evaluated, because citations might steadily increase with growing research age. An extreme example would be that a researcher terminates his/her work abruptly due to job change, end of the career or sudden death, while his/her *h*-index can theoretically still increase. Nevertheless, this increase would then obviously no longer reflect the researcher's current performance. Therefore, the *h-*index is of particular importance when it comes to assessing an individual's lifetime achievement or if researchers of similar research age should be compared. The mere *h*-index as such, without taking the research age into account, is, yet, of limited value for predicting one's potential future research impact (cf. [11], [12], [13], [14]). As the future of any research program, in fact, is also based on the high-quality continuation of research by young scientists, it is of relevance to use a standard tool for providing such predictions. If we were be able to predict future research impact already at an early stage we would be in the comfortable situation to also compare persons at different stages of their careers, increasing the chance of involving also highly talented young researchers in promising research streams.

### The *Carbon_h*-factor

To be compatible with the established and widely used *h*-index, the main logic of the here developed and proposed *Carbon_*factor is based on this index. It integrates, however, also the research age of the analyzed researcher. Research age versus biological age seems a much more straightforward predictor given the fact that any career, promising or not, can be started at any period of life [8]. In accord, biological age does not seem to be very tightly linked with publication impact [15]. Furthermore, research age can be easily retrieved by consulting the citation distribution over the years itself, whereas the biological age is often not publicly accessible, and can thus neither automatically nor with certainty be retrieved. Also, the *h*-index itself is inherently linked to the research age, as the first increase of the *h*-index can be prompted as a result of the first publication enlisted in the ISI Web of Science (accessible via http://isiknowledge.com), which would then mark the “year 0” of one's research career. By relating the *h-*index to the research age (research_age) the *Carbon_h*-factor is calculated as follows:
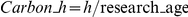
To take the factors of age into account of assessing one's performance is not new (e.g., [8], [11]). Most work in this respect, however, focused on biological age [e.g.], [ 15,16], a relatively weak predictor of scientific impact (e.g., [15]), versus the more significant research age. Additionally, the integration of research age has not yet been addressed in a systematic way.

The present paper analyzes the validity and stability of this index for research impact, based on ideas already developed only months after Hirsch's seminal paper on the *h*-index by Liang and followers [11], [12], by conducting a couple of analyses. In a pre-study, it will be shown that the *h-*index steadily increases even when an analyzed researcher has not published one single paper after a certain point of time, for instance because he or she died. The main study analyzes how the *h-*index of researchers performing on different research impact levels develops over time. Typical researchers' developments are important to appraise the validity of a single measurement predicting future research impact. The *Carbon_h*-factor seems valuable as a predictor for research impact only if a clear and stable relationship between research age and the *h*-index emerges.

## Methods and Materials

### Pre-study: analysis of the development of the *h*-index for researchers whose career ended immediately

The pre-study was conducted to analyze how research impact defined on basis of the *h-*index develops when a researcher's career ends right after it begins, for instance because the researcher dies.

Due to the logic of this paper, details on the methods are found in the method section of the main study below.

#### Research impact

Just as in the main study described, we were interested in the research impact defined in terms of developments of the *h-*index [4] over a researcher's lifetime. We selected only research careers which showed an intermediate termination of their research efforts, for instance as a result of the researcher's death. Four different research areas (economics, neuroscience, physics and psychology) on two different performance levels (substantial and outstanding) were taken into account. The performance level *epochal*, an important part of the main study, could not be integrated as hardly any cases pertaining to this performance level and fulfil the criterion of immediate termination of their career are reported in the scientific databases. For each of these 4 [research areas]×2 [performance levels] = 8 data cells, developments of two different researchers' outputs were analyzed in total yielding 16 individual research tracks.

#### Apparatus

The same as in the main study.

#### Procedure

The same procedure was used as described in the main study with the exception that only data of *substantial* and *outstanding* performers were calculated. For all researchers, the *h-*index for every year of their careers was calculated. On average this figure was 30.7 research years (range: 23–34 years), which is highly comparable with the values obtained in the main study.

### Main study: calculating the *Carbon_h*-factor for a variety of research areas

The main study aimed to assess the quality of the *Carbon_h-*factor, particularly its predictive quality. To extend the validity of this study, research impact data from four different research areas were used (economics, neuroscience, physics and psychology). In order to assess typical ranges *Carbon_h*-factors for differently performing researchers and to be able to validly predict future research impact, three different levels of high performance were analyzed, referred to as *substantial*, *outstanding* and *epochal* performance. All in all, for each of the four research areas and three levels of performance, ten different researchers' outputs were analyzed to reduce noise from idiosyncratic developments of their careers.

#### Research impact

Research impact defined in terms of developments of the *h-*index [4] over a researcher's lifetime were analyzed. This was done for four different research areas (economics, neuroscience, physics and psychology) on three different performance levels (substantial, outstanding and epochal). For each of these 4 [research areas]×3 [performance levels] = 12 data cells, developments of ten different researchers' outputs were analyzed in total yielding 120 individual research tracks. Although in the following only research age is used as an indicator of age, it should be noted that the researchers' mean years of birth of the respective research areas were 1953.8, 1953.1, 1952.2 and 1952.3, respectively, indicating highly compatible ages among the researchers within the different research disciplines. This was also the case when research age was used. The mean numbers of years that could be tracked for their scientific career were 26.4, 29.2, 32.4 and 28.5, which indicates that the researchers' profiles and their data in terms of research impact were also quite comparable.

#### Apparatus

As retrieval tool for the *h-*indices the ISI Web of Science was used comprising the following databases: 1) “Science Citation Index Expanded (SCI-EXPANDED) [1899-present]”, 2) “Social Sciences Citation Index (SSCI) [1899-present], 3) Arts & Humanities Citation Index (A&HCI) [1975-present], 4) New Conference Proceedings Citation Index- Science (CPCI-S) [1994-present], and 5) New Conference Proceedings Citation Index- Social Science & Humanities (CPCI-SSH) [1994-present]. The retrieval was conducted in 2010 for the period from 1975–2008 [inclusive].

#### Procedure

First, the absolute top performers of every single research area based on the sum of citations accumulated over the duration of the researchers' careers documented in the HighlyCited section of ISI Web of Knowledge, provided by Thomson Reuters, were determined. This accounted for highly different numbers with physics performing at the highest citation level, followed by neuroscience, psychology and economics (see Table 1), which is quite compatible with a recent analysis comparing the *h*-index across different fields of research [15]. We decided to take the performance levels of these top performers (called *epochal* performers) as basis for selecting all other performance levels to obtain adequate performance levels taking the typical research impact within these fields into account (see Table 1). For all retrievals we considered only researchers that started to publish between 1975 and 1989 and still published in the period between 2008 and 2010. Both criteria only referred to any publications listed in the Web of Knowledge.

**Table 1:** Selection criteria for the different researchers' performance levels in terms of the four different research areas (in parentheses the respective names of areas in the ISI Web of Knowledge are given) of overall accumulated citations.

	economics	neuroscience	physics	Psychology
	(economics/business)	(neurosciences)	(physics)	(psychology/psychiatry)
**Substantial**	100–250	450–1,125	600–1,500	250–625
**Outstanding**	400–1,000	1,800–4,500	2,400–6,000	1,000–2,500
**Epochal**	2,000+	9,000+	12,000+	5,000+

Generally, *substantial* performers account for 5%–12.5% and *outstanding* performers for 20%–50% of *epochal* researchers' sum of citations.

On the basis of the found ranges of the top performers we selected ten performers for each research area and called them *epochal*.

Second, further researchers defined as performing at the levels *substantial* and *outstanding* were identified based on the following criteria: on the one hand, their research impact again had again to start between 1975 and 1989 and had to still be visible in the period between 2008 and 2010, on the other hand they had to have 5%–12.5% or 20%–50% of the research impact of the epochal performers within their fields (see Table 1).

For all researchers, the *h-*index for every year of their careers was calculated, on average *h*-indices covered a period of 30.1 research years (range: 21–35 years).

## Results and Discussion

### Pre-study: analysis of the development of the *h*-index for researchers whose career ended immediately

**Figure 1:**
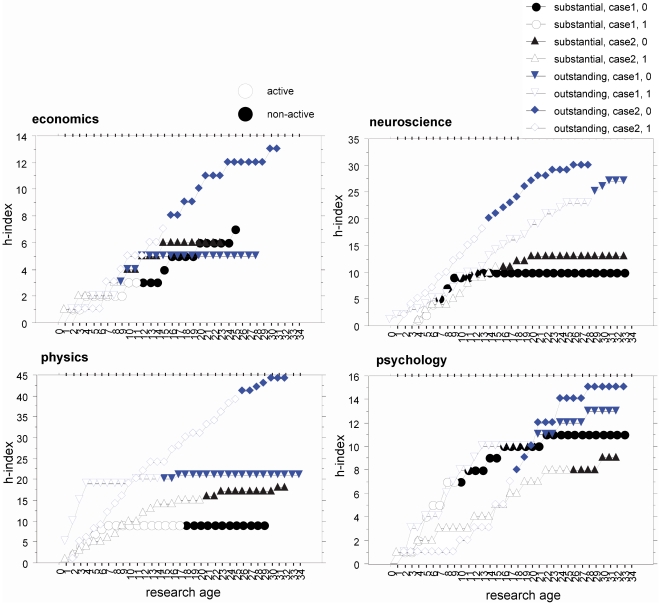
Pre-study. Calculation of the development of *h*-indices for careers that were immediately terminated, for instance, due to death of the researcher (“substantial” research impact is indicated in black, “outstanding” impact in blue). For each of the four research areas two cases per performance levels were analyzed. Non-solid dots indicate *h*-indices in the active era; solid dots show the data for the period following a researcher's active career. Note: Research age is defined within the realm of this paper on a pure technical basis starting at 0 coinciding with the year of the first publication relevant for the ISI Web of Science. From this moment on the research age increases every year by 1, independently of the researcher's lifetime, which means the research age increases even after the researcher has already passed away.

The *h*-index reflects the *n* of papers with at least *n* citations, thus every researcher's index is ultimately limited to the total number of papers that a researcher has published. As all researchers tracked in the pre-study had to exhibit a complete termination of their publication activity at some stage of their careers, the *h*-index is logically limited to the number of publications that were published before the end of their careers [12]. Figure 1 obviously shows that the development of many researchers' *h*-index, however, continues for a relatively long time rather constantly and converges to a saturation level only at a very late stage. For the selected researchers from the field of psychology this even holds true on the *substantial* as well as the *outstanding* performance level; even more impressive is case #2 of the *outstanding* performance level in the research area of economics, where the researcher's *h-*index reached 7 after having been active for 15 years, but nearly doubled after the same number of inactive years (Figure 1).

The overall pattern of results shows that the *h-*index increases in all but one case (physics, case#1 for the substantial performance level), for some researchers even continuously to a large degree, even after their active careers end (for instance due to the researcher's death). Thus, without taking the research age into account, any comparisons among researchers solely based on the absolute *h*-index might be invalid as a measure of research activity.

### Main study: calculating the *Carbon_h*-factor for a variety of research areas

**Figure 2:**
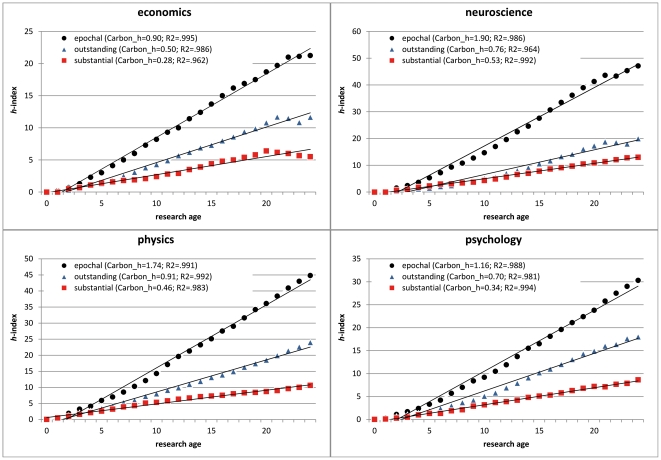
Main study. Development of *h-*indices for the four research areas split by the three different performance levels with increasing research age. The slope of the linear regression indicates the *Carbon_h-*factors, respectively. Curve fittings are based on aggregated data.

As researchers differ in the year of their first ISI Web of Science relevant publication, the statistical series differ in terms of research age. To be able to analyze on the one hand the longest possible research age ranges but on the other hand to base these analyses also on a minimum of missing values, we decided to limit the research age to the number of years accounting for at least 80% of occupied data cells. This criterion was met for 25 years of research age (0–24 years) covering still 80.8% of the researchers' full range of 25 research years. Figure 2 shows the development of researchers' *h*-index for the four different research areas performing at the three different levels. Already from Table 1, where we defined the criteria for the performance levels, it is clear that research areas strongly differ in their research impact which is due to combined effects of different publication strategies, level of competition, number of publication outlets, future orientation and financial equipment [3], [17], and, not to forget, the mere number of potential readers and citers [5], [18], [19]. The areas with the highest research impact were neuroscience and physics, followed by psychology and lastly economics. The whole dataset is provided in CSV (comma separated values) format, thus processable by all common software products via http://www.experimental-psychology.de/material_SOM.html.

**Figure 3:**
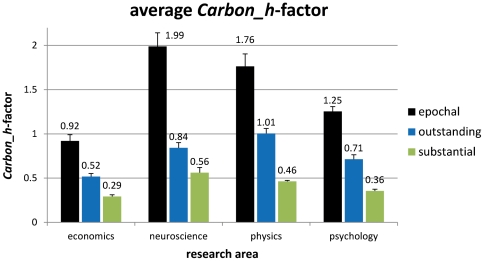
Main study. Average *Carbon_h*-factor for all four research areas on all three performance levels based on single linear curve fits of researchers' individual *h-*index developments over 25 years of research age. Error bars indicate standard errors of the mean.

One striking fact is that for all research areas at all performance levels tight linear relationships with the research age were revealed, *R* ^2^s>.962, highly compatible with earlier studies linking age and scientific performance [11]. Even if we calculated these correlations on individual data level, we gained very high Pearson's *R*s (Table 2 and Figure 3; *R*s>.883). This means that predictions of research impact even on basis of the existing data at a relatively early stage of a career are quite promising. In fact, we can observe a tight linear trend from approximately 10 years of research age on, in some disciplines for some performance levels even much earlier.

**Table 2:** Main study.

	economics	neuroscience	physics	psychology
**substantial**	0.29 [0.21–0.37] (*r* = .884)	0.56 [0.30–0.92] (*r* = .895)	0.46 [0.39–0.49] (*r* = .904)	0.36 [0.28–0.49] (*r* = .891)
**outstanding**	0.52 [0.33–0.67] (*r* = .916)	0.84 [0.59–1.13] (*r* = .883)	1.01 [0.77–1.27] (*r* = .920)	0.71 [0.51–0.99] (*r* = .918)
**epochal**	0.92 [0.56–1.21] (*r* = .945)	1.99 [1.21–2.94] (*r* = .938)	1.76 [1.08–2.70] (*r* = .950)	1.25 [1.10–1.64] (*r* = .928)

Mean *Carbon_h*-factors for the four research areas for each of the three performance levels. Ranges of *Carbon_h*-factors are given in parentheses in the second row. Furthermore, mean correlations (calculated as inverted Fisher-*Z*s on basis of individual correlations) between research age and *h*-index are given in the respective third row. Each of the cells contains *n* = 10 cases.

Figure 3 also gives the mean *Carbon_h* for each research area and all performance levels and Table 2 outlines additional statistics for these calculations including correlations between research age and *h-*index. Here, again, clear differences between the different research areas are visible, with substantial researchers' average *Carbon_h-*factor ranging between 0.29 and 0.56, outstanding researchers' *Carbon_h* between 0.52 and 1.01 and epochal researchers' *Carbon_h* between 0.92 and 1.99. Table 2, therefore, also provides a valuable source of decision basis as to which performance level an individual research activity is most probably to be assigned to.

**Figure 4:**
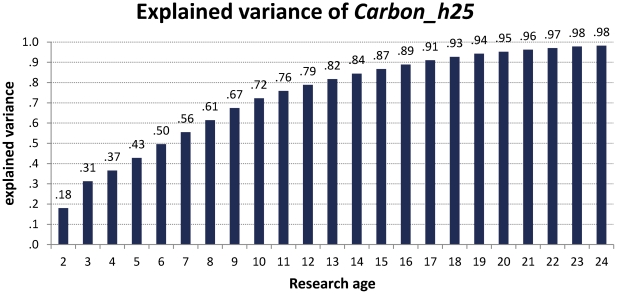
Main study. Comparing the *Carbon_h*-factor at several stages of research age from 2–24 compared with the *Carbon_h*-factor calculated at a research age of 25 (*Carbon_h*25), here expressed as *R* ^2^, the amount of explained variance.

To assess the predictive quality of the *Carbon_h* at certain stages of a career more elaborately, we employed an additional analysis in which the *Carbon_h-*factors were calculated based on different periods of research age. Starting with an inclusion of only the first 2 years up to 24 years of research age, we compared these *Carbon_h-*factors with the calculated ones of above, which is based on a research age of 25 years (*Carbon_h25*). When these *Carbon_h*-factors were calculated for all 120 researchers and correlated via Pearson *R* with the criterion *Carbon_h25*, we obtained a continuous approximation of explained variance ranging from *R* ^2^ = .18 up to .98 (Figure 4). Already after 10 years, the predictive quality of the respective *Carbon_h* was quite high explaining 72% of the variance of the *Carbon_h25*-factor. This replicates the above results regarding the developments of *h-*indices (Figure 2) that already at a research age of about 10 years we can predict the future research impact quite successfully.

To test the transferability of the found results to younger cohorts, an additional test was conducted with relatively young researchers having started their career between 1993 and 1999. These researchers were qualified by the selection criteria of Table 1 on the basis of predictive impact at research age 25 as *epochal* and *outstanding* researchers; only researchers from the areas of neuroscience and psychology were selected for these analyses. By using the same methodology and retrieval tools highly compatible outcome *Carbon_h-*factors were obtained for the 2 [research impact]×2 [research areas]×2 = 8 researchers (mean research age: 13.5 ys; range: 10–16 ys). When the maximum research age was limited to 10 years, which was the minimal research age range of the youngest researcher, the *Carbon_h*'s were 1.53 (*R* ^2^ = .983) and 1.11 (*R* ^2^ = .981) for neuroscience and 1.61 (*R* ^2^ = .917) and 0.57 (*R* ^2^ = .947) for psychology. This underlines the general compatibility of the here developed approach of predicting research impact at early stages of careers with a linear model also for younger researchers.

### General Discussion

The usage of objective indicators measuring research impact, such as the impact factor [20] or the *h*-index [4] has become a standard in evaluating individuals' careers, research programs or publication sources in terms of quality and quantity. It is generally disputable whether quality can be validly assessed by quantitative measures [5], [19], [21] and such indices are particularly susceptible to biases.

One major factor potentially promoting the *h*-index is the research age. To take the factors of age into account of assessing one's performance is not new (e.g., [8], [11]). Most work in this respect focused on the biological age (e.g., [15], [16]), a relative weak predictor of research impact (e.g., [15]), but not on the more relevant research age. Additionally, the integration of research age has not been yet addressed in a systematic way. As shown by the pre-study, the research age alone, independent of being highly active in research or being successful in producing publications, can explain most of the level of the *h*-index once a career is relatively advanced. With the examples of an extreme case of career development characterised by the sudden termination of all publication activities, for example, due to the researcher's death, the pre-study was able to show that *h-*indices can still continuously increase (see Figure 1) concealing recently gained true research merits.

The main study revealed for all research areas covered by the present study (economics, neuroscience, physics and psychology), which represent a wide spectrum of different research orientations, clear linear trends for the development of the *h*-index for three different levels of performance which were labelled as *substantial*, *outstanding* and *epochal*. This holds true for curve fits employed for aggregated as well as individual data underlining the linearity of career developments when measured by the *h-*index. By empirically testing such developments, not only the linear nature as such can be observed, but also the slope of the respective functions can be calculated and used for predictive reasons. The four targeted research areas differ substantially in terms of the slope of the linear function which we termed *Carbon_h*-factor, with neuroscience and physics showing the fastest increase of the *h-*index over the research age, followed by psychology and, lastly, by the field of economics and business administration. Following this empirical evidence, any qualification of a researcher's output should be adjusted to the specific field of research she or he works in just as proposed for the interpretation of the impact factor the *Carbon_h*-factor is indirectly based on [22]. On the empirical grounds of the development data of 120 single careers' *h*-indices (see Table 2) such rules of thumb would identify *substantial* researchers as having an annual increase of the *h*-index between 0.29–0.56 (overall mean: *M* = 0.42), *outstanding* researchers with an annual gain between 0.52–1.01 (*M* = 0.77) and *epochal* researchers with an annual achievement between 0.92–1.99 (*M* = 1.48).

Still, two important issues have to be addressed regarding the use of the *Carbon_h*-factor. First of all, the predictive quality clearly increases the longer the analyzed period of research age lasts. Nevertheless, as can be observed in Figure 2 and in statistical terms in Figure 4, the predictive quality is already very high when only the first ten years of research age are used to calculate the *Carbon_h*-factor. This is a quite promising fact and encourages the usage of the index at a relatively early stage of one's career. Importantly, the revealed tight linear relationship between research age and research impact can also found for younger researchers, already gained a research oeuvre of at least 10 years, as documented by the additional analysis of research careers started between 1993 and 1999. Second, any kind of quantitative unidimensional variable, as the here developed *Carbon_h*-factor, is always susceptible to neglecting other important sources (e.g., [16]), evidence and proof of high research impact, as already discussed in the introduction. Therefore, using any such measure that predicts impact, output or impact of research is clearly limited and can never be more than a raw heuristic. We should furthermore never underestimate the richness, the innovativeness and the exceptional quality of some scientists who do not fit into any standard system of research evaluation. Sometimes such figures are the real reason for advancing methods, theories and technology, some of them impacting science earlier, some of them later. Nevertheless, it can help to evaluate and compare research impact across researchers at different stages of their careers. Especially when researchers at early stages of their careers apply for important research positions, this could be of essential help to compare more mature and established researchers, and young, promising, and, most importantly, researchers with high potential.
